# Supplementary material for: Multi‐dataset identification of innovative feature genes and molecular mechanisms in keratoconus
Source: J Cell Mol Med. 2024 Sep 19;28(18):e70079. doi: 10.1111/jcmm.70079 (PMC11412914; doi:10.1111/jcmm.70079)
Supplement: Supplementary file 2 — Material S2. [file JCMM-28-e70079-s002.zip › 7.GSEA analysis/1.ARL11/KEGG_MISMATCH_REPAIR.html]

Details for gene set KEGG\_MISMATCH\_REPAIR[GSEA]

|  || Dataset | ARL11.group.cls#h\_versus\_l |
| Phenotype | group.cls#h\_versus\_l |
| Upregulated in class | h |
| GeneSet | KEGG\_MISMATCH\_REPAIR |
| Enrichment Score (ES) | 0.25643972 |
| Normalized Enrichment Score (NES) | 0.9133637 |
| Nominal p-value | 0.57973737 |
| FDR q-value | 1.0 |
| FWER p-Value | 1.0 |
Table: GSEA Results Summary

  

Fig 1: Enrichment plot: KEGG\_MISMATCH\_REPAIR      
 Profile of the Running ES Score & Positions of GeneSet Members on the Rank Ordered List

  

| SYMBOL | TITLE | RANK IN GENE LIST | RANK METRIC SCORE | RUNNING ES | CORE ENRICHMENT || 1 | RPA4 | na | 5183 | 0.160 | -0.0022 | Yes |
| 2 | RFC3 | na | 7177 | 0.108 | 0.0393 | Yes |
| 3 | RPA1 | na | 10886 | 0.049 | -0.0228 | Yes |
| 4 | MSH3 | na | 11101 | 0.046 | 0.0137 | Yes |
| 5 | PMS2 | na | 11257 | 0.045 | 0.0501 | Yes |
| 6 | POLD1 | na | 11636 | 0.040 | 0.0758 | Yes |
| 7 | PCNA | na | 11742 | 0.039 | 0.1085 | Yes |
| 8 | MLH3 | na | 13269 | 0.025 | 0.0872 | Yes |
| 9 | SSBP1 | na | 14376 | 0.016 | 0.0699 | Yes |
| 10 | POLD4 | na | 16051 | 0.003 | 0.0248 | Yes |
| 11 | MSH2 | na | 16407 | 0.001 | 0.0154 | Yes |
| 12 | LIG1 | na | 16850 | -0.002 | 0.0048 | Yes |
| 13 | RPA2 | na | 18069 | -0.011 | -0.0204 | Yes |
| 14 | POLD3 | na | 18840 | -0.016 | -0.0280 | Yes |
| 15 | EXO1 | na | 19062 | -0.017 | -0.0185 | Yes |
| 16 | MSH6 | na | 21102 | -0.032 | -0.0477 | Yes |
| 17 | MLH1 | na | 22834 | -0.048 | -0.0533 | Yes |
| 18 | RFC5 | na | 23651 | -0.058 | -0.0237 | Yes |
| 19 | RPA3 | na | 23802 | -0.060 | 0.0268 | Yes |
| 20 | POLD2 | na | 24003 | -0.062 | 0.0782 | Yes |
| 21 | RFC4 | na | 25422 | -0.082 | 0.1123 | Yes |
| 22 | RFC2 | na | 25466 | -0.082 | 0.1866 | Yes |
| 23 | RFC1 | na | 25828 | -0.088 | 0.2564 | Yes |
Table: GSEA details [plain text format]

  

Fig 2: KEGG\_MISMATCH\_REPAIR      
 Blue-Pink O' Gram in the Space of the Analyzed GeneSet

  

Fig 3: KEGG\_MISMATCH\_REPAIR: Random ES distribution      
 Gene set null distribution of ES for **KEGG\_MISMATCH\_REPAIR**
